# Long-Term Prestress Loss Calculation Considering the Interaction of Concrete Shrinkage, Concrete Creep, and Stress Relaxation

**DOI:** 10.3390/ma16062452

**Published:** 2023-03-19

**Authors:** Weiwei Han, Panpan Tian, Yigang Lv, Chaosheng Zou, Tao Liu

**Affiliations:** 1 National Engineering Research Center of Highway Maintenance Technology, Changsha University of Science & Technology, Changsha 410114, China; 2 School of Traffic & Transportation Engineering, Changsha University of Science & Technology, Changsha 410114, China; 3 School of Civil Engineering, Changsha University of Science & Technology, Changsha 410114, China

**Keywords:** loss of prestress, field test, creep by contraction, relaxation of stress, time-varying, law model

## Abstract

In order to accurately calculate the long-term prestress losses of prestressed tendons, a time-varying model of long-term prestress loss considering the interaction between concrete shrinkage, creep, and the stress relaxation of prestressed tendons was constructed. Then, a method for calculating the long-term prestress losses of concrete structures was developed. A long-term prestress loss test of a prestressed concrete T-beam in a long-term field test environment was carried out. The measured values of long-term prestress losses are compared with the calculated results of JTG 3362-2018, AASHTO LRFD-2007, and the time-varying law model. The results show that the long-term effective tension of the T-beam decreases gradually with the increase in the load holding time. At the beginning of loading, the tensile force changes rapidly and then gradually slows down. The later the tensile age or the higher the initial loading stress level, the smaller the long-term prestress losses of the prestressed tendons. The long-term prestress loss values calculated by JTG 3362-2018, AASHTO LRFD-2007, and the time-varying law model increase with the increase in the load holding time. In the early stage of loading, the rate of change slows down and tends to be stable. The calculated results of JTG 3362-2018 and AASHTO LRFD-2007 are significantly different from the measured values. However, the calculated results of the time-varying law model are in good agreement with the measured values. The average coefficients of variation of the long-term prestress loss calculated by JTG 3362-2018, AASHTO LRFD-2007, and the time-varying law model are 17%, 10%, and 5%, respectively. The time-varying law model of the long-term prestress losses of prestressed tendons is accurate, and the long-term prestress loss of prestressed reinforcement can be predicted effectively.

## 1. Introduction

Prestress loss is the key factor affecting the cracking of prestressed concrete bridges under torsion; it can be divided into short-term prestress loss and long-term prestress loss. Short-term prestress loss mainly includes the prestress loss caused by the friction between the prestressed bar and the pipe wall, anchor deformation, retraction of the steel bar, etc., which is easy to measure and calculate because it is independent of time [1,2,3,4]. The long-term prestress loss caused by prestressed rib relaxation, concrete shrinkage, and concrete creep is time-sensitive and difficult to calculate [5,6,7,8,9,10]. Long-term prestress loss accounts for more than 30% of the total prestress loss, and it has a great influence on the long-term performance of concrete bridge structures [11,12,13,14]. Therefore, determining how to accurately calculate the long-term prestress losses of prestressed tendons is of great significance.

Scholars at home and abroad have conducted some theoretical and experimental studies on the long-term prestress losses of concrete structures. Lu et al. [15] developed a long-term prestress loss calculation method for concrete structures based on the Latin hypercube sampling method. The effective modulus method was adjusted by age based on accurate and rapid sampling. It was also applied to the calculation of the prestress loss of a C50 concrete test beam, and the measured value of the long-term prestress loss of the test beam was located in the middle of the confidence interval of the prestress loss calculated using this method. Pablo M. Páez et al. [16] proposed a simplified equation for improving the prediction of the long-term prestress loss of unbonded prestressed concrete components considering concrete shrinkage creep, the stress relaxation of prestressed ribs, and the influence of bonded non-prestressed ribs. Compared with the simplified existing model, the proposed equation can fully predict the prestress loss with higher accuracy. Guo et al. [17] used load cells, vibrating line strain gauges (VWSG), and elastic magnetic (EM) sensors to measure prestress losses due to the creep and shrinkage of concrete, as well as the total prestress losses. After obtaining the long-term prestress loss of post-tensioned concrete beams, an improved model for predicting time-varying prestress loss is proposed. Compared with the existing model, the accuracy of the results is improved, and the maximum difference between the test results and the predicted results is within 10%. Based on their test results and for ease of calculation, Cao et al. [18] used the least squares method to fit the long-term prestress losses. Samer et al. [19], using equilibrium and compatibility principles based on solid mechanics, presented an analytical method for predicting the long-term prestress losses of precast, pre-tensioned, or post-tensioned concrete members. It can be used for multi-stage loading and prestressing. Yang et al. [20] used the median integral theorem based on the shrinkage creep models of FIB MC 2010 and AASHTO-LRFD 2014 to create a refined method for estimating the time-varying prestress loss. Compared with the numerical results obtained by the step-by-step method, it has good accuracy. Zhang et al. [21] established a finite element analysis (FEA) model based on the long-term prestress loss test of prestressed concrete beams and used the ABAQUS UMAT software to establish and calibrate it. Combined with an artificial neural network (ANN), a long-term prestress loss prediction model is proposed. Compared with the measured results, the prediction model is more accurate and efficient in the long-term prestress loss assessment of a prestressed concrete cylinder structure. The Chinese JTG 3362-2018 specification [22] adopts the itemized overlay method. Specifically, the losses caused by shrinkage and creep and the losses caused by the prestressed tendon stress relaxation of concrete are calculated separately and then added together to obtain the total loss. The US AASHTO-LRFD 2007 specification [23] considers the interactions between long-term prestress losses. The losses caused by time-dependent concrete shrinkage and creep, as well as the stress relaxation of prestressed tendons, are calculated separately. Finally, the total losses are obtained by adding them together.

The long-term prestress losses caused by concrete shrinkage and creep, as well as prestressed rib stress relaxation, have a mutual influence, as well as time-varying and uncertain changes over time [24,25,26]. In the calculation of prestress loss, the total effect of the interaction between concrete shrinkage, creep, and prestressed rib stress relaxation is compared with the effect considering their influences alone. The deviation value is generally large and cannot be ignored. At present, there are few studies on the calculation method of long-term prestress loss considering the interaction between the two in the design codes and the literature. As a result, the calculation results are quite different from the actual situation [27,28,29,30]. The long-term performance of concrete structures is an uncertain variable associated with the environment. However, most of the relevant experimental studies were carried out indoors, which is quite different from the complex natural environments in which concrete structures are located [31,32]. To accurately calculate the long-term prestress losses of concrete structures, a long-term time-varying model of prestress loss considering the interaction between concrete shrinkage, creep, and the stress relaxation of prestressed tendons is proposed. In addition, a long-term prestress loss test of a prestressed concrete T-beam in a long-term field test environment was carried out, and a long-term prestress loss calculation method for concrete structures was formed for test verification. It made the calculation of the long-term prestress loss of the structure safer and more reliable.

## 2. Calculation Method of Long-Term Prestress Loss Considering the Interaction of Shrinkage, Creep, and Stress Relaxation

### 2.1. Basic Assumptions

In the process of deducing the formula of long-term prestress loss, the following basic assumptions are satisfied [33,34,35]:

1. At any time, the elastic stress and elasticity of concrete should become linear;
2. The assumption of flat section deformation of the beam body is valid;
3. The creep deformation is linear, satisfying the superposition principle;
4. Ordinary reinforcement and prestressed reinforcement are completely bonded to the concrete without slip;
5. There is no cracking phenomenon in the concrete section.

### 2.2. Relaxation of Prestressed Reinforcement

When calculating the instar ($t_{i}$) moment, the inherent relaxation loss ($\sigma_{\chi }\left(t_{i}\right)$) of prestressed reinforcement is calculated as follows [33]:

(1) $$\sigma_{\chi }\left(t_{i}\right)=\frac{\sigma_{p0}}{10}\cdot \left(\frac{\sigma_{p0}}{C\cdot f_{py}}-0.55\right)\cdot log\left(t_{i}-t_{p}+1\right)$$

where *t_p_* is the tensile age of the prestressed tendons (d); $\sigma_{p0}$ is the effective prestress of the force transmission anchorage; $f_{py}$ is the standard value of the tensile strength of the prestressed tendons; and C is the constant related to steel (0.85 for general steel and 0.90 for low-slack steel).

During the loading period from $t_{0}$ to $t_{i}$, the variation in the natural relaxation loss of prestressed tendons ($\sigma_{l}\left(t_{i},t_{0}\right)$) is

(2) $$\sigma_{l}\left(t_{i},t_{0}\right)=\sigma_{\chi }\left(t_{i}\right)-\sigma_{\chi }\left(t_{0}\right)$$

When $t_{0}$=$t_{p}$, $\sigma_{\chi }\left(t_{0}\right)=0$.

In the actual prestressed concrete structure, there is no constant strain. Using inherent relaxation as stress loss would overestimate the loss. In the concrete unit, due to the influence of concrete shrinkage and creep, the relaxation of prestressed tendons is less than their inherent relaxation. Therefore, the reduction in the relaxation loss of prestressed tendons must be used in the design calculation ($\bar{\sigma_{l}}\left(t_{i},t_{0}\right)$):

(3) $$\bar{\sigma_{l}}\left(t_{i},t_{0}\right)=\lambda \left(t_{i}\right)\sigma_{l}\left(t_{i},t_{0}\right)$$

where $\lambda \left(t_{i}\right)$ is the stress relaxation reduction coefficient from $t_{0}$ to $t_{i}$, considering the effects of shrinkage and creep.

### 2.3. Time-Varying Law Model of Long-Term Prestress Loss

**Figure 1:**
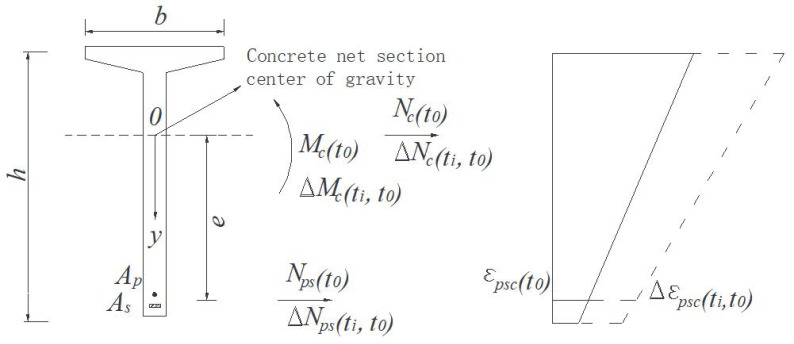
Calculation of prestress losses of prestressed concrete beams.

When calculating the long-term prestress loss of the prestressed reinforcement of a prestressed concrete beam under the interactive influences of shrinkage, creep, and prestress relaxation, the interaction of the precompression zone and the pre-tension zone is ignored [36,37,38,39]. A calculation diagram of the prestress loss of a concrete beam is shown in Figure 1.

In Figure 1, the areas of non-prestressed and prestressed tendons are $A_{s}$ and $A_{p}$; the distance between the center of gravity and the center of gravity of the net section of concrete is e; the net area and the net moment of inertia of the concrete section are $A_{n}$ and $I_{n}$; at the loading age $t_{0}$, the normal force and bending moment of the concrete section on the section are $N_{c}\left(t_{0}\right)$ and $M_{c}\left(t_{0}\right)$; the normal force of the rebar is $N_{ps}\left(t_{0}\right)$; and the concrete strain at the center of the rebar is $\varepsilon_{psc}\left(t_{0}\right)$. 

During the period from $t_{0}$ to $t_{i}$, the external load does not change. In order to simplify the calculation, the centers of gravity of prestressed reinforcement and non-prestressed reinforcement in the prestressed zone (or pre-stretching zone) are approximately concentrated on the two centers of gravity. Then,

(4) $$\{\begin{array}{l} \Delta \varepsilon_{p}\left(t_{i},t_{0}\right)=\Delta \varepsilon_{psc}\left(t_{i},t_{0}\right)\\ \Delta \varepsilon_{s}\left(t_{i},t_{0}\right)=\Delta \varepsilon_{psc}\left(t_{i},t_{0}\right) \end{array}$$

where $\Delta \varepsilon_{psc}\left(t_{i},t_{0}\right)$, $\Delta \varepsilon_{p}\left(t_{i},t_{0}\right)$, and $\Delta \varepsilon_{s}\left(t_{i},t_{0}\right)$, respectively, represent the strain increment of concrete, prestressed reinforcement, and non-prestressed reinforcement at the center of gravity of reinforcement during the period from $t_{0}$ to $t_{i}$.

Considering the inherent relaxation of the reinforcement without strain change, the relationship between the stress increment and the strain increment of each reinforcement is as follows:

(5) $$\{\begin{array}{l} \Delta \sigma_{p}\left(t_{i},t_{0}\right)=E_{p}\Delta \varepsilon_{p}\left(t_{i},t_{0}\right)+\lambda \left(t_{i}\right)\sigma_{l}\left(t_{i},t_{0}\right)\\ \Delta \sigma_{s}\left(t_{i},t_{0}\right)=E_{s}\Delta \varepsilon_{s}\left(t_{i},t_{0}\right) \end{array}$$

where $\Delta \sigma_{s}\left(t_{i},t_{0}\right)$ and $\Delta \sigma_{p}\left(t_{i},t_{0}\right)$ are the stress increment of non-prestressed reinforcement and prestressed reinforcement, respectively, and $E_{s}$ and $E_{p}$ are the elastic moduli of non-prestressed and prestressed tendons, respectively.

According to Equations (4) and (5), the internal force change ($\Delta N_{ps}\left(t_{i},t_{0}\right)$) of prestressed reinforcement and non-prestressed reinforcement in the period from $t_{0}$ to $t_{i}$ is

(6) $$\Delta N_{ps}\left(t_{i},t_{0}\right)=\left(A_{p}+\frac{E_{s}}{E_{p}}A_{s}\right)E_{p}\Delta \varepsilon_{psc}\left(t_{i},t_{0}\right)+\lambda \left(t_{i}\right)\sigma_{l}\left(t_{i},t_{0}\right)A_{p}$$

Within the period from $t_{0}$ to $t_{i}$, the increment in the normal force ($\Delta N_{c}\left(t_{i},t_{0}\right)$) on the concrete part of the section and the increment in the bending moment ($\Delta M_{c}\left(t_{i},t_{0}\right)$) on the barycenter axis of the net section of the concrete are

(7) $$\{\begin{array}{l} \Delta N_{c}\left(t_{i},t_{0}\right)=-\Delta N_{ps}\left(t_{i},t_{0}\right)\\ \Delta M_{c}\left(t_{i},t_{0}\right)=\Delta N_{c}\left(t_{i},t_{0}\right)\cdot e \end{array}$$

The concrete stress increment ($\Delta \sigma_{psc}\left(t_{i},t_{0}\right)$) at the center of gravity of reinforcement ($\rho_{s}=A_{s}/A_{n}$, $\rho_{p}=A_{p}/A_{n}$, $r_{n}=\sqrt{I_{n}/A_{n}}$, and $\rho_{ps}=1+e^{2}/r_{n}^{2}$) is

(8) $$\Delta \sigma_{psc}\left(t_{i},t_{0}\right)=\frac{\Delta N_{c}\left(t_{i},t_{0}\right)}{A_{n}}+\frac{\Delta M_{c}\left(t_{i},t_{0}\right)}{I_{n}}\cdot e\;=-\left[\left(\rho_{p}+\frac{E_{s}}{E_{p}}\rho_{s}\right)\cdot E_{p}\Delta \varepsilon_{psc}\left(t_{i},t_{0}\right)+\rho_{p}\lambda \left(t_{i}\right)\sigma_{l}\left(t_{i},t_{0}\right)\right]\cdot \rho_{ps}$$

According to the principle of linear superposition, under a constant load, the concrete strain increment ($\Delta \varepsilon_{psc}\left(t_{i},t_{0}\right)$) at the center of gravity of reinforcement is as follows when it is loaded for a longer time than $t_{0}$ ($t_{i}$> $t_{0}$):

(9) $$\Delta \varepsilon_{psc}\left(t_{i},t_{0}\right)=\frac{\sigma_{psc}\left(t_{0}\right)\cdot \phi \left(t_{i},t_{0}\right)}{E_{c}\left(t_{0}\right)}+\frac{\Delta \sigma_{psc}\left(t_{i},t_{0}\right)\cdot [1+\chi \left(t_{i},t_{0}\right)\cdot \phi \left(t_{i},t_{0}\right)]}{E_{c}\left(t_{0}\right)}+\varepsilon_{sh}\left(t_{i},t_{0}\right)\;=\frac{\sigma_{psc}\left(t_{0}\right)\cdot \phi \left(t_{i},t_{0}\right)}{E_{c}\left(t_{0}\right)}-n_{p}\rho_{ps}\left(\rho_{p}+\frac{E_{s}}{E_{p}}\rho_{s}\right)\cdot [1+\chi \left(t_{i},t_{0}\right)\cdot \phi \left(t_{i},t_{0}\right)]\Delta \varepsilon_{psc}\left(t_{i},t_{0}\right)\;-\frac{\rho_{p}\rho_{ps}[1+\chi \left(t_{i},t_{0}\right)\cdot \phi \left(t_{i},t_{0}\right)]\lambda \left(t_{i}\right)\sigma_{l}\left(t_{i},t_{0}\right)}{E_{c}\left(t_{0}\right)}+\varepsilon_{sh}\left(t_{i},t_{0}\right)$$

where $E_{c}\left(t_{0}\right)$ and $\sigma_{psc}\left(t_{0}\right)$ are, respectively, the elastic modulus and the initial stress of the concrete at time $t_{0}$; $\phi \left(t_{i},t_{0}\right)$ refers to the creep coefficient with a loading age of $t_{0}$ and calculating the creep coefficient with a loading age of $t_{i}$; $\chi \left(t_{i},t_{0}\right)$ is the aging coefficient; $\varepsilon_{sh}\left(t_{i},t_{0}\right)sh$ is the shrinkage strain of the concrete beam at time $t_{i}$; and $n_{p}=E_{p}/E_{c}\left(t_{0}\right)$. Among them, $\phi \left(t_{i},t_{0}\right)$ and $\varepsilon_{sh}\left(t_{i},t_{0}\right)sh$ were calculated according to reference [40].

According to Equations (4) and (9), we can obtain

(10) $$\Delta \varepsilon_{p}\left(t_{i},t_{0}\right)=1/\left\{1+n_{p}\rho_{ps}\left(\rho_{p}+\frac{E_{s}}{E_{p}}\rho_{s}\right)\cdot [1+\chi \left(t_{i},t_{0}\right)\cdot \phi \left(t_{i},t_{0}\right)]\right\}\cdot \{\frac{\sigma_{psc}\left(t_{0}\right)\cdot \phi \left(t_{i},t_{0}\right)}{E_{c}\left(t_{0}\right)}\;-\frac{\rho_{p}\rho_{ps}[1+\chi \left(t_{i},t_{0}\right)\cdot \phi \left(t_{i},t_{0}\right)]\lambda \left(t_{i}\right)\sigma_{l}\left(t_{i},t_{0}\right)}{E_{c}\left(t_{0}\right)}+\varepsilon_{sh}\left(t_{i},t_{0}\right)\}$$

From $t_{0}$ to $t_{i}$, the time-varying law model of the long-term prestress loss ($\Delta \sigma_{p}\left(t_{i},t_{0}\right)$) of prestressed tendons is

(11) $$\Delta \sigma_{p}\left(t_{i},t_{0}\right)=E_{p}\Delta \varepsilon_{p}\left(t_{i},t_{0}\right)+\lambda \left(t_{i}\right)\sigma_{l}\left(t_{i},t_{0}\right)\;=\left\{n_{p}\sigma_{psc}\left(t_{0}\right)\phi \left(t_{i},t_{0}\right)+E_{p}\varepsilon_{sh}\left(t_{i},t_{0}\right)+\left\{1+n_{s}\rho_{s}\rho_{ps}[1+\chi \left(t_{i},t_{0}\right)\cdot \phi \left(t_{i},t_{0}\right)]\right\}\cdot \lambda \left(t_{i}\right)\sigma_{l}\left(t_{i},t_{0}\right)\right\}/\left\{1+n_{p}\rho_{ps}\left(\rho_{p}+\frac{E_{s}}{E_{p}}\rho_{s}\right)\cdot [1+\chi \left(t_{i},t_{0}\right)\cdot \phi \left(t_{i},t_{0}\right)]\right\}$$

where it is equal to $n_{s}=E_{s}/E_{c}\left(t_{0}\right)$.

In the common range of prestressed concrete structures, $E_{s}\approx E_{p}$ can be approximated; the common range of the aging coefficient ($\chi \left(t_{i},t_{0}\right)$) is 0.6~0.9, while 0.82 is generally preferable; and the stress relaxation reduction coefficient ($\lambda \left(t_{i}\right)$) generally varies from 0.5 to 0.9. Generally, 0.75 is taken. The simplified time-varying law model of $\Delta \sigma_{p}\left(t_{i},t_{0}\right)$ is

(12) $$\Delta \sigma_{p}\left(t_{i},t_{0}\right)=\left[n_{p}\sigma_{psc}\left(t_{0}\right)\phi \left(t_{i},t_{0}\right)+E_{p}\varepsilon_{sh}\left(t_{i},t_{0}\right)\right]/\left\{1+n_{p}[1+0.82\phi \left(t_{i},t_{0}\right)]\rho \rho_{ps}\right\}\;+\left\{0.75\left\{1+n_{s}\rho_{s}\rho_{ps}[1+0.82\phi \left(t_{i},t_{0}\right)]\right\}\sigma_{l}\left(t_{i},t_{0}\right)\right\}/\left\{1+n_{p}[1+0.82\phi \left(t_{i},t_{0}\right)]\rho \rho_{ps}\right\}$$

where it is equal to $\rho =\left(A_{s}+A_{p}\right)/A_{n}$.

## 3. Long-Term Field Test

### 3.1. Experimental Materials

The cement in the raw material of the test concrete is ordinary Portland cement with a strength of 52.5 MPa, produced by China Gezhouba Group Cement Co., Ltd. The aggregate is produced from 5~25 mm basalt crushed stone from the Lianhuaqiao crushing field in Changsha County and river sand from Dongting Lake, and the steel bars and strands are produced by Hunan Valin Lianyuan Iron and Steel Co., Ltd. In order to ensure the accuracy of the test and reduce the interference of unfavorable factors in the test results, the aqueous states of the aggregates used in this test are all dry after natural air drying.

### 3.2. Test Setup

**Figure 2:**
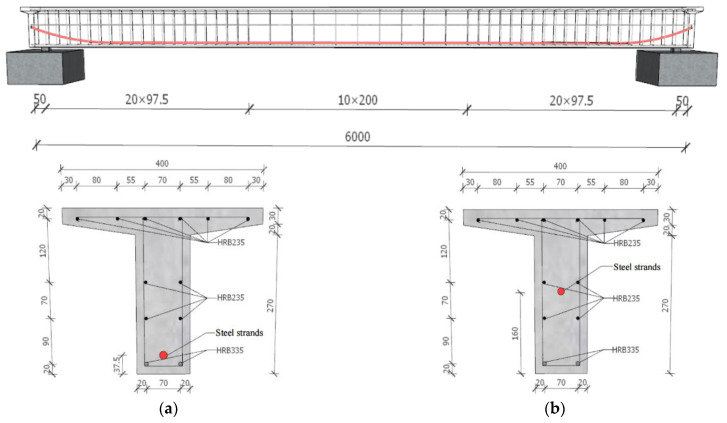
Size and reinforcement of T-beam (unit: mm): (**a**) mid-span section and (**b**) fulcrum cross-section.

**Figure 3:**
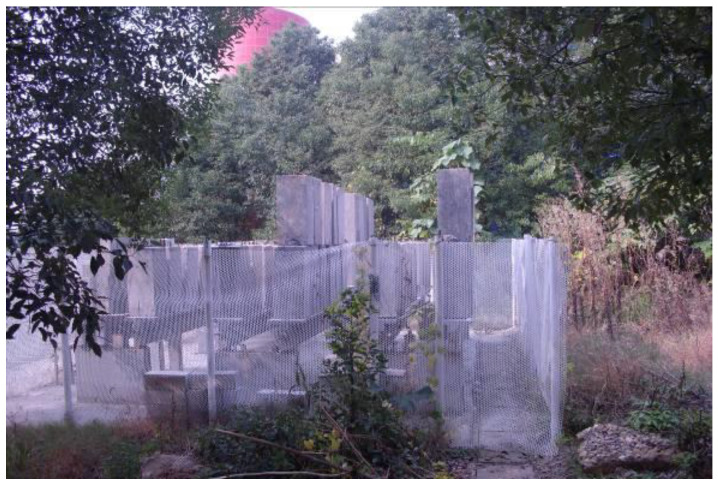
Natural maintenance of T-beam field test station.

A field test station was established in the living environment of the general structure, and a long-term prestress loss test of a prestressed concrete T-beam was carried out. A total of four pieces of bonded prestressed concrete T-beams with 6.0 m lengths were produced. Their numbers were B1#~B4#. The calculated span of the T-beams was 5.8 m, the beam height was 0.32 m, the flange plate width was 0.40 m, and the T-rib width was 0.11 m. The cement was 52.5# ordinary Portland cement, and the concrete mix ratio was cement/sand/gravel/water/water-reducing agent = 460:585:1175:232.5:3.68. The lower edge was equipped with two HRB335 longitudinal steel bars with diameters of 14 mm and one low-relaxation prestressed steel strand with a diameter of 15.24. The upper edge was equipped with six vertical R235 steel bars with diameters of 6 mm, as shown in Figure 2. The tensioned control stress ($\sigma_{con}$) of each beam was 1395 and was single-end tensioned. The tensioned age ($t_{p}$) of B1# and B2# was 7 d, and the tensioned age ($t_{p}$) of beams B3# and B4# was 28 d. The test T-beam was placed in the field test station for natural maintenance, as shown in Figure 3. The cubic compressive strength and elastic modulus of the concrete at an age of 28 d were 57.0 MPa and 36.7 GPa, respectively. Taking the average value of the previous 365 d as the annual average temperature and annual average humidity, the measured annual average temperature was approximately 23.80 °C, and the measured annual average relative humidity was approximately 64.2%.

### 3.3. Loading and Testing

**Figure 4:**
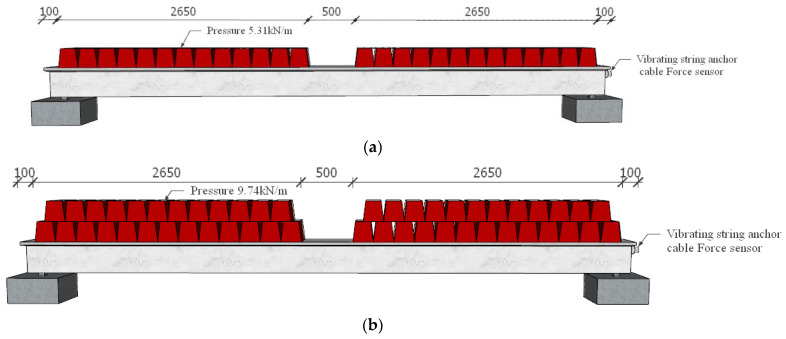
Loading of T-beam (unit: mm): (**a**) loading status of B1# and B3#; (**b**) loading status of B2# and B4#.

In the test, standard concrete blocks were locally uniformly loaded. The mechanical property parameters after loading are shown in Table 1. The loading mode of the test T-beam is shown in Figure 4. A vibrating wire strain gauge was embedded at one end of each test T-beam to test the effective prestress of the prestressed steel strand. 

## 4. Results and Discussion

### 4.1. Long-Term Effective Tensile Test Results and Analysis

**Figure 5:**
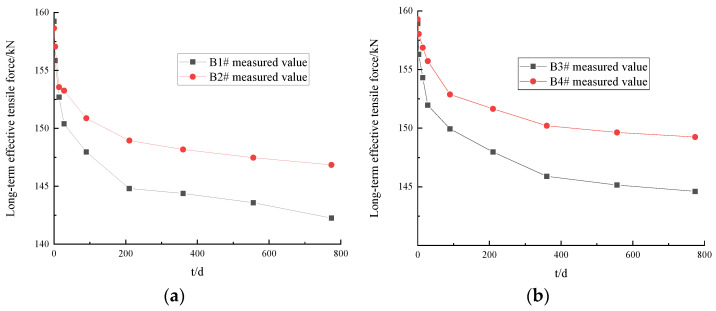
Results of long-term effective tensile test of test T-beam: (**a**) loading age = 7 d; (**b**) loading age = 28 d.

Taking the loading age $t_{0}$ as the starting time, the vibrating wire strain gauge embedded in the test T-beam was used to test the long-term effective tension of the prestressed tendons of B1#~B4# in the field test environment. The long-term effective tensile force values of the prestressed tendons of the T-beam varied in all tests with the load holding time (t), as shown in Figure 5.

As can be seen in Figure 5, the development trend of the long-term effective tensile force of the prestressed tendons of the T-beam was essentially the same in all tests. With an increase in the load holding time (t), the long-term effective tensile force gradually decreased. The tensile force changed rapidly in the initial stage of loading and gradually slowed down in the later stage. When the load holding time (t) was 774 d, the long-term tensile loss (ΔP) values of the prestressed tendons of B1#~B4# were 16.74 kN, 11.8 kN, 14.32 kN, and 10.04 kN, respectively. When the load holding time (t) was 90 days, the tensile loss values of the prestressed tendons of B1#~B4# were 66%, 65%, 62%, and 63% of those of ΔP, respectively. When the load holding time (t) increased from 90 d to 360 d, the tensile loss values of the prestressed tendons of B1#~B4# were 84%, 86%, 89%, and 89% of those of ΔP, respectively. 

### 4.2. Analysis of Influence of Tensile Age and Initial Loading Stress Level

(1) Tensile age

**Figure 6:**
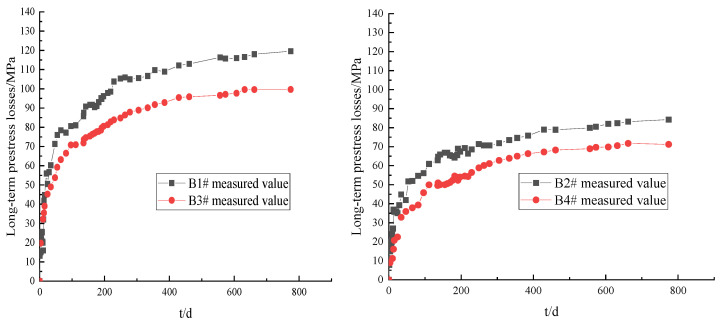
Comparison of long-term prestress losses at different tension ages.

The same initial loading stress level was maintained. Then, the long-term prestress loss values of prestressed tendons corresponding to B1# and B3#, and B2# and B4#, were compared at different tensile ages ($t_{p}$), as shown in Figure 6. 

As can be seen in Figure 6, the long-term prestress losses of the prestressed tendons of each test T-beam at different tensile ages gradually increased with the holding time. The early stage of development was relatively fast, and the later stage tended to gradually flatten. When the load holding time (t) was 774 d, the ratio of the long-term prestress losses of B1# and B3# was 1.169, and that of B2# and B4# was 1.175. The results show that, with the same initial loading stress level, the later the tensile age ($t_{p}$), the smaller the influence of concrete shrinkage and creep and the smaller the long-term prestress loss of the prestressed tendons.

(2) Initial loading stress level

**Figure 7:**
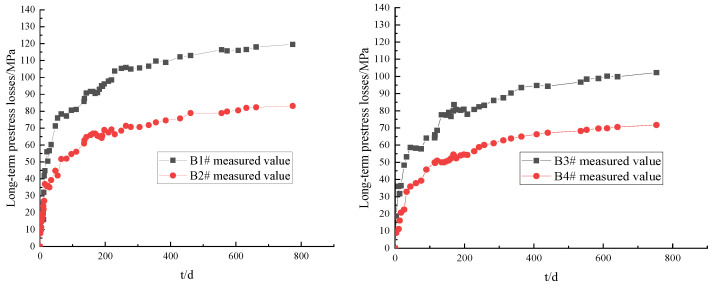
Comparison of long-term prestress loss values at different initial loading stress levels.

The same tensile age ($t_{p}$) was maintained, and the long-term prestress losses of the prestressed tendons corresponding to B1# and B2#, and B3# and B4#, at different initial loading stress levels were compared, as shown in Figure 7. 

As can be seen in Figure 7, the long-term prestress loss values of the prestressed tendons of each test T-beam with different initial loading stress levels gradually increased with the holding time. The early stage of development was relatively fast, and the later stage tended to gradually flatten. When the load holding time (t) was 774 d, the ratio of the long-term prestress losses of B1# and B2# was 1.418, and that of B3# and B4# was 1.425. The results show that, with the same loading age ($t_{p}$), the higher the initial loading stress level of the test beam, the smaller the long-term prestress loss of the prestressed tendons. 

### 4.3. Comparison and Verification of the Measured Values and Calculated Results

Taking the loading age of $t_{0}$ as the starting time, the long-term prestress losses of the prestressed tendons of beams 1#~4# in the field test environment were calculated using the JTG 3362-2018 specification [21], the AASHTO LRFD-2007 specification [22], and the long-term prestress loss time-varying law model of this paper. 

The calculation process of the JTG 3362-2018 specification is as follows.

(1) Prestress loss caused by relaxation of prestressed ribs:

(13) $$\sigma_{l5}=\psi \cdot \zeta \cdot \left(0.52\frac{\sigma_{pe}}{f_{py}}-0.26\right)\cdot \sigma_{pe}$$

where ψ is the tension coefficient; ψ = 1.0 with one tension; ψ = 0.9 with an over tensile state; ζ is the steel bar relaxation coefficient; for level I relaxation (ordinary relaxation), ζ = 1.0; for level II relaxation (low relaxation), ζ = 0.3; $\sigma_{pe}$ is the rebar stress (MPa) during anchoring.

(2) Prestress loss caused by concrete shrinkage creep:

(14) $$\sigma_{l6}\left(t\right)=\frac{0.9\left[E_{p}\varepsilon_{cs}\left(t,t_{0}\right)+\alpha_{Ep}\sigma_{pc}\phi \left(t,t_{0}\right)\right]}{1+12\rho \rho_{ps}}$$

where $\sigma_{l6}\left(t\right)$ is the prestress loss (MPa) caused by concrete shrinkage and creep at the center of gravity of the longitudinal rebar section; $\sigma_{pc}$ is the normal stress of concrete (minus the first loss) generated by the prestress of the center of gravity of all longitudinal rebar sections (MPa); $E_{p}$ is the modulus of elasticity (MPa) of the prestressed rebar; $\alpha_{Ep}$ is the ratio of the elastic modulus of prestressed steel bars to the elastic modulus of concrete; ρ is the longitudinal reinforcement ratio of the component; $\varepsilon_{cs}\left(t,t_{0}\right)$ is the concrete shrinkage strain at the computed age t, when the age of the transmitting of forces and anchorage of prestressed reinforcement is assumed as $t_{0}$; $\phi \left(t,t_{0}\right)$ is the creep coefficient when the loading age is $t_{0}$, and the computed age is t. $\rho_{ps}$ is the parameter related to the radius of rotation of the section i and the distance $e_{ps}$ from the cross section gravity of prestressed reinforcement and non-prestressed rebar in the tensioning zone for members to the gravity axis of the cross section of members.

Long-term prestress loss:

(15) $$\sigma_{l11}=\sigma_{l5}+\sigma_{l6}\left(t\right)$$

The calculation process of the AASHTO LRFD-2007 specification is as follows.

(1) Prestress loss caused by relaxation of prestressed ribs:

(16) $$\Delta f_{pR1}=\frac{f_{pt}}{K_{L}}\left(\frac{f_{pt}}{f_{py}}-0.55\right)$$

where $f_{pt}$ is the stress of the prestressed steel strand after the anchor is transmitted, and the value is not less than 0.55$f_{py}$ (MPa); if $K_{L}$ does not have more accurate factory data, we take 30 for a low-relaxation steel strand, and 7 for other prestressed steel bars.

(2) Prestress loss due to concrete shrinkage:

(17) $$\Delta f_{pSD}=\frac{\varepsilon_{bdf}E_{p}}{1+\frac{E_{p}A_{ps}}{E_{ci}A_{c}}\left(1+\frac{A_{c}e_{pc}^{2}}{I_{c}}\right)\left[1+0.7\psi_{b}\left(t_{f},t_{i}\right)\right]}$$

where $\varepsilon_{bdf}$ is the shrinkage strain of concrete from the moment of plate installation to the final moment; $e_{pc}$ is the eccentricity (mm) of the prestress shape of the combined section; $A_{c}$ is the cross-sectional area (mm^2^); $I_{c}$ is the cross-sectional moment of inertia (mm^4^). $A_{ps}$ is the area of prestressing steel. $E_{ci}$ is concrete bullet mold. $\psi_{b}\left(t_{f},t_{i}\right)$ is girder creep coefficient at final time due to loading introduced. $t_{f}$ is final age (days). $t_{i}$ is age at transfer (days).

(3) Prestress loss caused by concrete creep:

(18) $$\Delta f_{pCD}=\frac{E_{p}}{E_{ci}}f_{cgp}\left[\psi_{b}\left(t_{f},t_{i}\right)-\psi_{b}\left(t_{d},t_{i}\right)\right]K_{df}+\frac{E_{p}}{E_{c}}\Delta f_{cd}\psi_{b}\left(t_{f},t_{d}\right)K_{df}$$

where $\Delta f_{cd}$ is the change in concrete stress at the center of the steel strand caused by long-term stress loss during the installation of the anchor to the plate, and the self-weight and applied load (MPa) of the plate are considered. $\psi_{b}\left(t_{f},t_{d}\right)$ is the creep coefficient of the final time component. $f_{cgp}$ is sum of concrete stresses at the center of gravity of prestressing tendons due to the prestressing force after jacking and the self-weight of the member at the sections of maximum moment(MPa). $K_{df}$ is transformed section coefficient that accounts for time-dependent interaction between concrete and bonded steel in the section being considered for time period between deck placement and final time. $\psi_{b}\left(t_{d},t_{i}\right)$ is girder creep coefficient at time of deck placement due to loading introduced. $t_{d}$ is age at deck placement (days).

Long-term prestress loss:

(19) $$\Delta f_{pLT}=\Delta f_{pSD}+\Delta f_{pCD}+\Delta f_{pR1}$$

**Figure 8:**
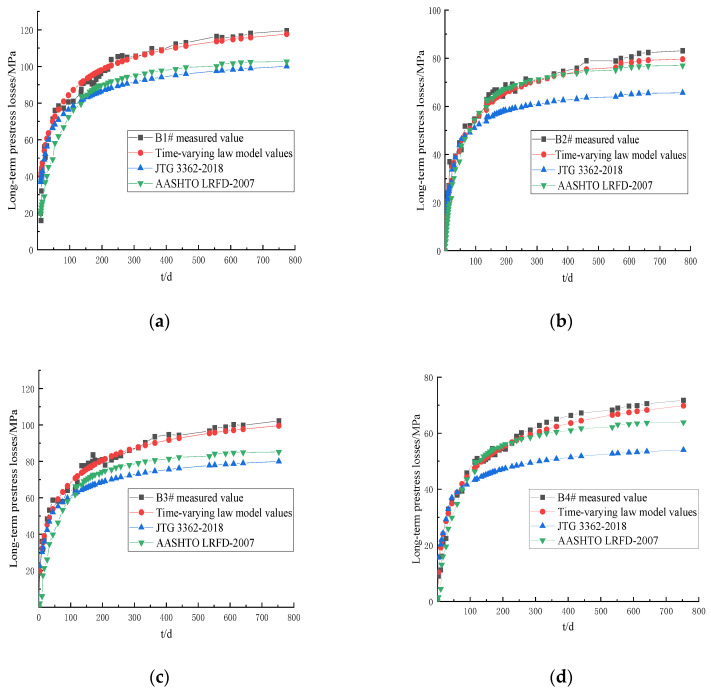
Comparison of the measured values of the long-term prestress losses of the test T-beams and the calculation results: (**a**) B1#; (**b**) B2#; (**c**) B3#; (**d**) B4#.

The comparison between the measured values and the calculated results of the long-term prestress losses of the prestressed tendons of the T-beam in each test at different load holding times (t) is shown in Figure 8.

As can be seen in Figure 8, the calculated results of the long-term prestress losses of the prestressed tendons using the JTG 3362-2018 specification, the AASHTO LFD-2007 specification, and the time-varying law model were essentially consistent with the development trend of the measured values. With an increase in the load holding time (t), the long-term prestress losses gradually increased. The long-term prestress losses developed rapidly in the initial loading stage. In the later period, the rate of change gradually slowed down and tended to be stable. When the load holding time (t) was less than 100 d, the calculated results of the three models were in good agreement with the measured values. However, as the load holding time (t) continued to increase, the differences between the calculated results of JTG 3362-2018 and AASHTO LFD-2007 and the measured values became larger and larger. When the load holding time (t) was 774 d, the relative errors of long-term prestress loss calculated by JTG 3362-2018 for B1#~B4# were 1.198, 1.272, 1.281, and 1.327, respectively. The relative errors of long-term prestress loss calculated by the AASHTO LRFD-2007 code were 1.164, 1.094, 1.200, and 1.072, respectively. The relative errors of long-term prestress loss calculated by the time-dependent law model were 1.017, 1.039, 1.027, and 1.027, respectively. The error between JTG 3362-2018 and AASHTO LRFD-2007 was large. The calculated results of the long-term prestress losses of the prestressed tendons using the time-varying law model were in good agreement with the measured values.

The calculated results of the long-term prestress losses of the prestressed tendons using JTG 3362-2018, AASHTO LRFD-2007, and the time-varying law model had variation coefficients (ϑ) relative to the measured values of each test T-beam, as shown in Table 2.

Remarks: $\vartheta =\sqrt{\frac{1}{n-1}\sum_{i=1}^{n}{\left(\Delta \sigma_{p,i}^{’}-\Delta \sigma_{p,i}\right)}^{2}}/\bar{\Delta \sigma_{p}}$, $\bar{\Delta \sigma_{p}}=\sum_{i=1}^{n}\Delta \sigma_{p,i}/n$, and $\bar{\vartheta }=\sqrt{\frac{1}{n}\sum_{j}\vartheta^{2}}$. $\Delta \sigma_{p,i}^{’}$ is the calculated value of the long-term prestress loss (i); $\Delta \sigma_{p,i}$ is the measured value of the long-term prestress loss (i); ϑ is the j group of the variation coefficient, and $\bar{\vartheta }$ is the coefficient of variation relative to all the data.

As can be seen in Table 2, the average coefficients of variation of JTG 3362-2018, AASHTO LRFD-2007, and the time-varying law model were 17%, 10%, and 5%, respectively. The results show that the interaction of concrete shrinkage, concrete creep, and stress relaxation is considered in the theoretical derivation, and more accurate calculation results are obtained.

## 5. Conclusions

- The interaction between concrete shrinkage, creep, and the stress relaxation of prestressed reinforcement was considered. A time-varying model of the long-term prestress losses of prestressed concrete beams was established. A calculation method for the long-term prestress losses of concrete structures was developed. The calculation results of the long-term prestress loss of a structure were more secure and reliable.
- A long-term prestress loss test of a prestressed concrete T-beam in a long-term field test environment was carried out. The development trend of the long-term effective tensile force of the prestressed tendons of the T-beam was essentially the same in all tests. With an increase in the load holding time, the long-term effective tensile force gradually decreased. The tensile force changed rapidly in the initial stage of loading and gradually slowed down in the later stage. When the load holding time was 774 d, the long-term tensile losses of the prestressed tendons of B1#~B4# were 10.52%, 7.44%, 9.01%, and 6.30%, respectively.
- The influence of the tensile age and the initial loading stress level on the long-term prestress losses of prestressed concrete T-beams was analyzed. When the holding time was 774 d, the ratios of the long-term prestress losses of beams 1# and 3# and beams 2# and 4# were 1.169 and 1.175, respectively. This shows that the later the tensile age, the smaller the long-term prestress loss of the prestressed tendons. The ratios of the long-term prestress losses of beams 1# and 2# and beams 3# and 4# were 1.418 and 1.425, respectively. This indicates that the higher the initial loading stress level, the smaller the long-term prestress loss of the prestressed tendons.
- The measured values of the long-term prestress losses of the prestressed concrete T-beams were compared with the calculated results of the JTG 3362-2018 specification, the AASHTO LRFD-2007 specification, and the time-varying law model. The calculated results of the long-term prestress losses of the three theoretical models were essentially consistent with the development trend of the measured values. When the load holding time was less than 100 d, the calculated results of the three models were in good agreement with the measured values. However, as the load holding time continued to increase, the differences between the calculated results of JTG 3362-2018 and AASHTO LRFD-2007 and the measured values became larger and larger. The average coefficients of variation of the long-term prestress loss calculation results of JTG 3362-2018, AASHTO LRFD-2007, and the time-varying law model were 17.23%, 10.29%, and 5.64%, respectively. This shows that the time-varying law model of the long-term prestress losses of prestressed tendons established in this study has good accuracy and can better predict the long-term prestress losses of prestressed tendons.

In this paper, a long-term time-varying model of prestress loss considering the interaction of concrete shrinkage, concrete creep, and stress relaxation is proposed. The long-term prestress loss test of a prestressed T-beam under a long-term exposure test environment was carried out for test verification. A long-term prestress loss calculation method for concrete structures was formed, which makes the calculation of the internal forces of bridge structures safer and more reliable.

## Figures and Tables

**Table 1:** Mechanical property parameters of the secondary loading of the T-beam.

Number of Beam	Locally Uniformly Distributed Load		Dead Weight + Local Uniform Load + Prestress	
	Value of Load (kN/m)	Tensile Age (*t*_0_/d)	Upper-Edge Concrete Stress (MPa)	Lower-Edge Concrete Stress (MPa)
B1#	5.31	7	2.64	4.84
B2#	9.74	7	6.34	−0.42
B3#	5.31	28	2.64	4.84
B4#	9.74	28	6.34	−0.42

**Table 2:** The coefficient (ϑ ) of variation of each predictive model relative to the measured values (unit: %).

Model of Prediction	B1#	B2#	B3#	B4#	Mean Coefficient of Variation
JTG 3362-2018	15.69	17.27	17.93	20.15	17
AASHTO LRFD	12.56	8.73	10.54	9.34	10
Time-varying law model	9.10	4.88	4.12	4.98	6

## Data Availability

Data are contained within the article.

## References

[1] Pavlović A., Donchev T., Petkova D., Limbachiya M. (2022). Short- and Long-Term Prestress Losses in Basalt FRP Prestressed Concrete Beams under Sustained Loading. J. Compos. Constr..

[2] Xu Q., Zeng B., Xu X.D., Wang X.F. (2022). Distribution Characteristics and Estimation Method of Prestress in Concrete Structures Based on Gaussian Mixture Model. J. Archit. Struct..

[3] Zheng X., He M., Lam F., Sun X., Liang F., Li Z. (2022). Experimental and Numerical Investigation of Long-Term Loss of Prestressing Force in Posttensioned Timber Joints with Different Structural Details. J. Struct. Eng..

[4] Shokoohfar A., Rahai A. (2017). Prediction model of long-term prestress loss interaction for prestressed concrete containment vessels. Arch. Civ. Mech. Eng..

[5] Moravčík M., Kraľovanec J. (2022). Determination of Prestress Losses in Existing Pre-Tensioned Structures Using Bayesian Approach. Materials.

[6] Tahsiri H., Belarbi A. (2022). Evaluation of prestress relaxation loss and harping characteristics of prestressing CFRP systems. Constr. Build. Mater..

[7] Gao X., Jia J., Mei G., Bao X., Zhang L., Liao X. (2022). A New Prestress Loss Calculation Model of Anchor Cable in Pile–Anchor Structure. Mathematics.

[8] Biswal S., Ramaswamy A. (2017). Uncertainty based model averaging for prediction of long-time prestress losses in concrete structures. Constr. Build. Mater..

[9] Sun G., Li Z., Wu J., Qu X., Ren J. (2022). Investigation into the Prestress Loss and Thermal Expansion Performance of Steel Cables at High Temperature. Int. J. Steel Struct..

[10] Lee S., Lee C. (2022). Bonding Time and Prestress Loss in Precast Pretensioned Concrete during Steam Curing. J. Struct. Eng..

[11] Kim S.H., Park S.Y., Kim S.T., Jeon S.J. (2022). Analysis of Short-Term Prestress Losses in Post-tensioned Structures Using Smart Strands. Int. J. Concr. Struct. Mater..

[12] Kim S.T., Park Y.S., Yoo C.H., Shin S., Park Y.H. (2021). Analysis of Long-Term Prestress Loss in Prestressed Concrete (PC) Structures Using Fiber Bragg Grating (FBG) Sensor-Embedded PC Strands. Appl. Sci..

[13] Abdel-Jaber H., Glisic B. (2019). Monitoring of long-term prestress losses in prestressed concrete structures using fiber optic sensors. Struct. Health Monit..

[14] Bonopera M., Chang K.C., Lee Z.K. (2020). State-of-the-art review on determining prestress losses in prestressed concrete girders. Appl. Sci..

[15] Lu Z.F., Liu M.Y. (2011). A Calculation Method for Long-term Prestress Loss of Concrete Structures. J. Wuhan Univ. Technol..

[16] Páez P.M., Sensale B. (2018). Improved prediction of long-term prestress loss in unbonded prestressed concrete members. Eng. Struct..

[17] Guo T., Chen Z., Lu S., Yao R. (2018). Monitoring and analysis of long-term prestress losses in post-tensioned concrete beams. Measurement.

[18] Cao G., Zhang W., Hu J., Zhang K. (2018). Experimental study on the long-term behaviour of RBPC T-beams. Int. J. Civ. Eng..

[19] Samer A., Youakim A.G., Hida S.E. (2007). Prediction of long-term prestress losses. PCI J..

[20] Yang M., Gong J., Yang X. (2020). Refined calculation of time-dependent prestress losses in prestressed concrete girders. Struct. Infrastruct. Eng..

[21] Zhang H., Guo Q., Xu L.Y. (2023). Prediction of long-term prestress loss for prestressed concrete cylinder structures using machine learning. Eng. Struct..

[22] CCCC Highway Consultants Co, Ltd (2018). Code for Design of Highway Reinforced Concrete and Prestressed Concrete Bridges and Culverts: JTG 3362-2018.

[23] (2009). AASHTO LRED Bridge Design Soecifications (2007).

[24] Pan Z.F., Lv Z.T., Liu Z. (2009). Uncertainty Analysis of Shrinkage and creep Effect of Continuous Rigid Frame on Sutong Bridge. Eng. Mech..

[25] Bazant Z.P. (1972). Prediction of concrete creep effects using age-adjusted effective modulus method. ACI J..

[26] Bažant Z.P. (2002). Concrete fracture models: Testing and practice. Eng Fract Mech..

[27] Alghazali H., Myers J. (2017). Time-dependent prestress loss behavior of girders in Missouri bridge A7957 compared with a US data set of high-performance concrete bridge girders. PCI J..

[28] Guo T., Ping Z., De L., Tian Z. (2021). Study on short-term prestress loss of bridge reinforced with large diameter carbon fiber bars. IOP Conf. Ser. Earth Environ. Sci..

[29] Shi K., Wu X., Tian Y., Xie X. (2021). Analysis of Re-Tensioning Time of Anchor Cable Based on New Prestress Loss Model. Mathematics.

[30] Li X., Deng J., Wang Y., Xie Y., Liu T., Rashid K. (2021). RC beams strengthened by prestressed CFRP plate subjected to sustained loading and continuous wetting condition: Time-dependent prestress loss. Constr. Build. Mater..

[31] Bellendir E.N., Rubin O.D., Lisichkin S.E., Zyuzina O.V. (2021). Experimental Study into Prestress Losses of Basalt Composite Reinforcement Used in the Composition of Concrete Elements. Power Technol. Eng..

[32] Yue S., Chu Q. (2020). Prestress Loss Analysis and Overload Early Warning Research of Simply Supported Girder Bridge Based on Embedded Computer and Fiber Grating Sensing Technology. J. Phys. Conf. Ser..

[33] Kamatchi R., Rao K.B., Dhayalini B., Saibabu S., Parivallal S., Ravisankar K., Iyer N. (2014). R Long-Term Prestress Loss and Camber of Box-Girder Bridge. ACI Struct. J..

[34] Li G., Wu J.T. (2022). Stress relaxation characteristics of prestressed GFRP anchor under erosion conditions. Chin. J. Rock Mech. Eng..

[35] Xu F., Li D., Zhang Z., Zhang Q.T., Kang L., Wei Y. (2019). Comparison of Concrete Creep-Induced Structural Prestress Loss Calculated by Codes from Different Countries. J. Highw. Transp. Res. Dev..

[36] Shi J., Wang X., Wu Z., Wei X., Ma X. (2022). Long-term mechanical behaviors of uncracked concrete beams prestressed with external basalt fiber-reinforced polymer tendons. Eng. Struct..

[37] Sung D., Hong S. (2022). Experimental study on long-term behavior of prestressed steel I-beam-concrete composite beams. Steel Compos. Struct..

[38] Dai L., Bian H., Wang L., Potier-Ferry M., Zhang J. (2020). Prestress loss diagnostics in pretensioned concrete structures with corrosive cracking. J. Struct. Eng..

[39] Chai S., Guo T., Chen Z., Yang J. (2019). Monitoring and simulation of long-term performance of precast concrete segmental box girders with dry joints. J. Bridge Eng..

[40] Han W., Lü Y. (2016). Experimental research on prediction model of concrete shrinkage and creep. J. Cent. South Univ. (Sci. Technol.).

